# Further Insight into the Depth-Dependent Microstructural Response of Cartilage to Compression Using a Channel Indentation Technique

**DOI:** 10.1155/2013/358192

**Published:** 2013-04-03

**Authors:** Ashvin Thambyah, Neil D. Broom

**Affiliations:** Tissue Mechanics Laboratory, Department of Chemical and Materials Engineering, University of Auckland, Private Bag 92019, Auckland 1142, New Zealand

## Abstract

Stress relaxation and structural analysis were used to investigate the zonally differentiated microstructural response to compression of the integrated cartilage-on-bone tissue system. Fifteen cartilage-on-bone samples were divided into three equal groups and their stress relaxation responses obtained at three different levels of axial compressive strain defined as low (*~*20%), medium (*~*40%) and high (*~*60%). All tests were performed using a channel indenter which included a central relief space designed to capture the response of the matrix adjacent to the directly loaded regions. On completion of each stress relaxation test and while maintaining the imposed axial strain, the samples were formalin fixed, decalcified, and then sectioned for microstructural analysis. Chondron aspect ratios were used to determine the extent of relative strain at different zonal depths. The stress relaxation response of cartilage to all three defined levels of axial strain displayed an initial highly viscous response followed by a significant elastic response. Chondron aspect ratio measurements showed that at the lowest level of compression, axial deformation was confined to the superficial cartilage layer, while in the medium and high axial strain samples the deformation extended into the midzone. The cells in the deep zone remained undeformed for all compression levels.

## 1. Introduction

The cartilage extracellular matrix consists primarily of heavily hydrated proteoglycan macromolecules constrained within a richly structured collagenous fibrillar network. The tissue resists load-induced deformation through both an intrinsic stiffness generated by the functional coupling between its high-swelling proteoglycans and constraining fibrillar network, and that arising from the resistance to fluid flow through its ultra low permeability structure. Both of these contributions are necessarily integrated in the matrix response to an applied load. Adding another level of complexity is the zonally differentiated structure of the cartilage matrix. The articular surface layer with its in-plane arrangement of fibrils creates a tangentially strain-limiting upper layer which, via a transition zone, blends into the radial mid- and deep zones. This deep zone is integrated structurally with the subchondral bone via the zone of calcified cartilage. The loading of cartilage involves the deformation response of this entire zonally differentiated structural system including the movement of its fluid component. How such a complex structural system would respond, in a zonally integrated manner, to loading remains underexplored. 

There have been several published studies that have contributed to improving our understanding of how the microstructure of cartilage responds to compression. Studies of the depth-dependent response of cartilage to mechanical loading have shown that with increasing compressive strain, stress-relaxation proceeds at a reduced rate [1], and the average apparent modulus increases significantly [2, 3]. At the pericellular and cellular levels Choi et al. [4] showed that cartilage tissue compression resulted in a varying decrease in chondron height across the tangential, mid- and deep zones. The highest strains were found to occur in the tangential zone, followed by lesser amounts in the mid- and deep zones, and these changes increased with increasing levels of matrix compression [4]. 

The increased stiffening of cartilage with increasing compression, together with variations in deformation with zonal depth, is of significance to the modelling of cartilage mechanics. Using a biphasic model Wang et al. [5] modelled such inhomogeneity and showed how depth-dependent variations in the cartilage matrix stiffness resulted in corresponding changes in stress, strain, fluid velocity, and the fluid pressure field. However, the appreciation of models, in a material sense, is in some ways limited by the extent to which matrix deformation may be visualized, or how the physical fluid-solid interaction takes place within a theoretical framework. For example, with cartilage compression, the solid matrix strain-level-dependent microstructural response [6] fluid displacement through decreasing levels of matrix permeability [1], and distribution of loads away from directly compressed regions [7], are some intriguing physical realities that present major challenges to the modeller in attaining a truly accurate representation of cartilage mechanics. Some resolution of these challenges would be possible if the actual depth-dependent structural response of the tissue could be determined, especially in relation to the way loads are distributed. Recent developments by the present authors have attempted to address this structural-mechanical issue.

To date, two experimental procedures have been developed that have obvious relevance to this question of how the cartilage matrix and its coupled fluid respond to deformation [8, 9]. First is the method by which the compressed state of cartilage-on-bone samples is captured using chemical fixation, followed by decalcification and structural analysis using differential interference contrast (DIC) microscopy. This optical technique allows fully hydrated sections to be imaged at levels of structural resolution that are highly informative with regard to the pattern of matrix deformation [8]. Second is in the use of a compression indenter incorporating a central channel which creates two distinct sites of structural interest, namely, a directly loaded region and an indirectly loaded relief zone which develops within the channel space [10]. An analysis of the patterns of matrix deformation across these two sites provides new possibilities for investigating fluid flow-related effects, matrix permeability, fibrillar interconnectivity, and the role of the strain-limiting tangential layer. 

In this new study, we have utilised the previous experimental techniques to study the depth-dependent response of cartilage-on-bone to stress-relaxation following compression. In order to correlate the physical and mechanical behaviors, a straightforward application of a viscoelastic model was used to quantify the stress-relaxation response of cartilage and then compared with its microstructural deformation. 

## 2. Materials and Methods

### 2.1. Sample Preparation

Fifteen healthy bovine patellae obtained from 2-3-year-old prime bovine bulls were collected immediately following slaughter and stored at −20°C. For sample preparation each patella was thawed under cold running water, and the cartilage surface stained with India ink to confirm that there were no surface irregularities or fissures [11]. A cartilage-bone block with *en face* dimensions of ~14 × 14 mm was sawn from each distal-lateral quadrant whilst carefully selecting an optimally flat region. Each block included the full cartilage thickness and ~6 mm of subchondral bone to provide full loading support. Each of the four sides of the block was finely ground under water with 60-grit carborundum and imaged at low magnification to determine the average cartilage thickness. The block was then equilibrated in 0.15 M saline for two hours prior to loading, then secured in a custom-built stainless steel holder with dental cement.

### 2.2. Loading Protocol

**Figure 1:**
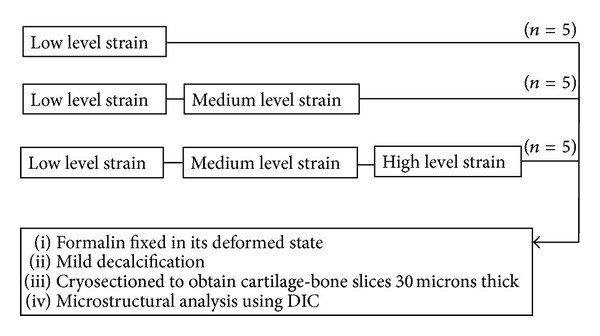
Sample testing and treatment procedures used.

All loading was performed using a specially designed polished flat-ended stainless steel indenter consisting of two flat 8 × 3 mm faces separated by a channel space of 1 mm and channel height of 3 mm. Details of this indenter have been described elsewhere [10]. An Instron 5543 materials testing machine was used for the stress relaxation experiments which were conducted with the samples bathed in 0.15 M saline. Three different loading/relaxation procedures were used as follows (Figure 1).

**Procedure 1**

Five samples were compressed to 20% strain at 1 mm/s and maintained in this constant state until a near-equilibrium stress state was reached as indicated by the force-time curve (time required, ~30 minutes).

**Procedure 2**

Five samples were loaded as in Procedure 1, then following the establishment of near-equilibrium, loaded a further 20% strain at 1 mm/s, and held in this state until a new near-equilibrium stress state was reached (~45 minutes).

**Procedure 3**

Five samples were loaded as in Procedure 2, followed by a third added level of compression of 20% strain, and held in this state until a new near-equilibrium stress state was reached (~60 minutes).

To avoid cartilage destruction, the three-step loading was applied. In preliminary tests direct loading to 40%, and at the 1 mm/s speed, had resulted in splitting the cartilage surface. Therefore with the 3-step loading protocol, with no observable cartilage damage occurring at the high strains, deep tissue compression was obtained in order to study the depth-dependent response reliably.

Following each loading protocol, after which near-equilibrium stress had been reached [1], the saline bath was replaced with 10% formalin and the sample fixed for ~12 hrs in its deformed state. 

### 2.3. Light Microscopy

Following the loading/fixation procedures described previously, each sample was washed in cold water to remove excess formalin and then mildly decalcified in 10% formic acid solution for 3 days (solution changed daily) to facilitate sectioning of the osteochondral region [8]. The sample was finally rinsed in cold running water for 30 minutes. 

A central radial cut was made through each decalcified osteochondral sample to obtain the full cross-sectional profile of the directly and nondirectly loaded matrix so as to incorporate the channel region and the wider cartilage continuum. The samples were then quick-frozen and cryo-sectioned using a sledging microtome to obtain 10–30 *μ*m thick frozen osteochondral sections close to the original radial cut. These sections were then wet mounted in saline on a glass slide under a cover slip and examined using (DIC) optical microscopy. After looking at multiple serial sections, and confirming their consistency, one typical section was set aside to represent each sample, giving a total of fifteen representative microsections.

### 2.4. Analysis of Deformation Field

As with our earlier work [8, 9] the chondrocytes were utilized as markers to capture the deformation response of the loaded matrix. The overall fibril orientation has been shown to be aligned with the long axis of the chondrocytes [12, 13]. Thus, by mapping the lines of chondrocyte continuity the generalised fibril orientation within the fields of deformation can be obtained. Further confirmation of the fibrillar arrangement was obtained by using high resolution DIC. Although individual fibrils were beyond optical resolution, their tendency to aggregate into larger bundles generates a directional fibrosity which again reflects overall fibrillar organisation [14–16]. 

**Figure 2:**
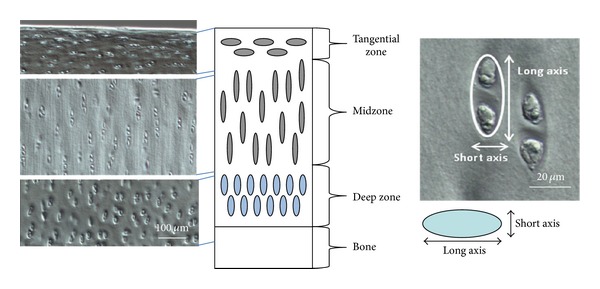
Chondron aspect ratio measurements obtained at different zonal depths. Typically, chondrons containing two chondrocytes were selected for each measurement.

The patterns of deformation were analysed in terms of changes in profiles and orientation of the lines of chondrocytes continuity. Chondron aspect ratios (Figure 2) were measured in the upper, mid- and deep zones of the cartilage matrix and in the directly compressed and channel relief regions as a means of determining differences in matrix compression with respect to zonal depth. All lengths were measured digitally from images imported into Image J digital image processing software.

### 2.5. Analysis of Stress Relaxation Behaviour

Nominal stress-time plots were obtained from the loading experiments. Peak and equilibrium stresses were obtained for each curve and their ratios calculated. The instantaneous stiffness response was used to estimate the initial modulus (MPa), calculated as the initial (maximum) stress divided by the imposed (constant) strain (low, medium, or high). 

A simple Maxwell model was used to quantify the relaxation response as follows:

(1) $$\begin{array}{c} \sigma \left(t\right)=Ae^{-t/\tau }, \end{array}$$

where the constant “*A*” is the peak stress before relaxation begins, that is, at *t* = 0 and constant strain, *ε*_0_, and “*τ*” is a time constant that determines the rate of decay of the stress. 

**Figure 3:**
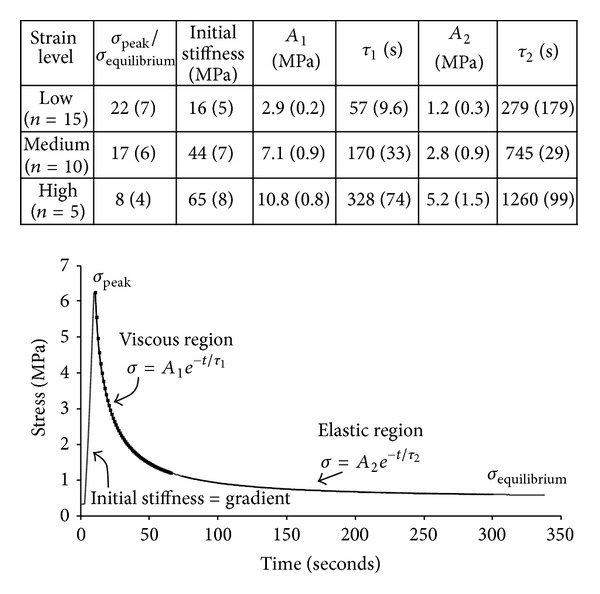
Typical stress relaxation curve obtained for a medium strain level compression test showing the viscous and elastic regions. The viscous response refers to the initial rapid stress decay, and the elastic response refers to the latter portion of the curve. The table inset summarises the parameters obtained from the curve fits from applying the Maxwell-body model to each region of the stress relaxation curve for the different strain levels tested. The criterion for each fit was *R* ^2^ > 0.9.

To ensure a satisfactory correlation between the Maxwell equation and the experimental data each relaxation curve was separated into an initial and a later response (see representative curve in Figure 3), the first representing the rapid stress decay (viscous) and the second the much slower relaxation response (relatively elastic). Each curve was thus approximated with two equations and two sets of parameters *A*, *τ* (in MPa and seconds, resp.). The curve fits for the data obtained from each test were applied manually; the observer used the “trendline” function in excel to check when the *R* ^2^ correlation would yield values of at least 0.9 in each region.

It should be emphasized here that our employment of the simple Maxwell model was not for the purpose of representing the complex mechanical behaviour of cartilage. Rather, it was utilized as a practical and convenient means of quantifying potential differences in mechanical behaviour and especially with regard to elastic and viscous responses. 

### 2.6. Statistical Analysis

Three groups of data were compiled for each of the low, medium, and high strain levels employed (i.e., low, medium, and high). The parameters, described in the analyses of the deformation field and stress relaxation behaviour above, were compared, and differences in their mean values between the three groups were tested for significance (*P* < 0.05) using a nonparametric Mann-Whitney statistical analysis.

## 3. Results

### 3.1. Mechanical Responses

**Figure 4:**
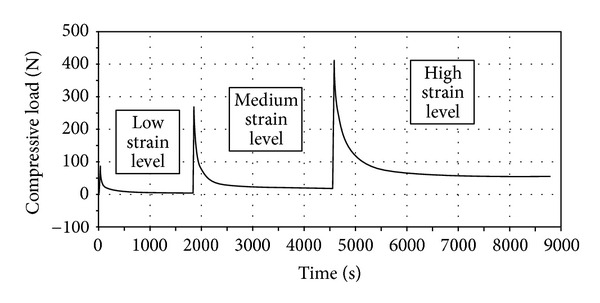
Typical force-time relaxation curves obtained following low, medium, and high levels of compressive strain.

A typical three-stage loading profile, covering the *low*, *medium,* and *high* strain levels, is shown in Figure 4. For the low strain level tests the mean initial modulus was 16 MPa (Standard Deviation), 5 MPa). The mean initial modulus for the medium and high levels of strain was significantly larger by about 175% and 44%, respectively (see table inset in Figure 3). The mean peak stress attained during the medium level compressive strain was also significantly larger than that obtained at the low strain level by about 170%. The increase in mean peak stress at the high strain level was 47% higher than that attained at the medium strain level. 

For all relaxation tests the final near-equilibrium stress was 0.2 MPa (SD, 0.09 MPa), 0.6 MPa (SD, 0.23 MPa), and 1.8 MPa (SD, 0.65 MPa) for the low, medium, and high level compressive strain levels, respectively. These three equilibrium values equated to an increase in equilibrium stress of ~200% between each level tested. 

With each added component of strain the ratio of peak to equilibrium stress decreased significantly (table inset in Figure 3). From the low to medium strain levels this ratio was reduced by ~22%, and from the medium to high strain levels it was reduced by ~53%. 

Each stress relaxation curve was divided into two regions defining the initial and end responses, such that fitting the Maxwell model would yield *R* ^2^ correlation values of at least 0.9 in each region (Figure 3). These regions were then used to approximate a mostly *viscous *response (fast rate of decay in stress) and a mostly *elastic *response (slow rate of decay of stress). The constants obtained from the curve fitting are shown (Table inset in Figure 3). 

In the viscous region there was a significant increase in *A* (an indicator of the shear or rigidity modulus) from the low-to-medium and medium-to-high levels of strain of 145% and 52%, respectively. For the elastic region *A* was overall lower than that for the viscous region, and increased 133% and 86% following low-to-medium and medium-to-high levels of strain, respectively. The relaxation times *τ* in the elastic region were significantly larger than in the viscous region. They also increased significantly from the low-to-medium and medium-to-high levels of strains in both the viscous (198% and 93%, resp.) and elastic regions (167% and 69%, resp.). 

### 3.2. Morphological Responses

**Figure 5:**
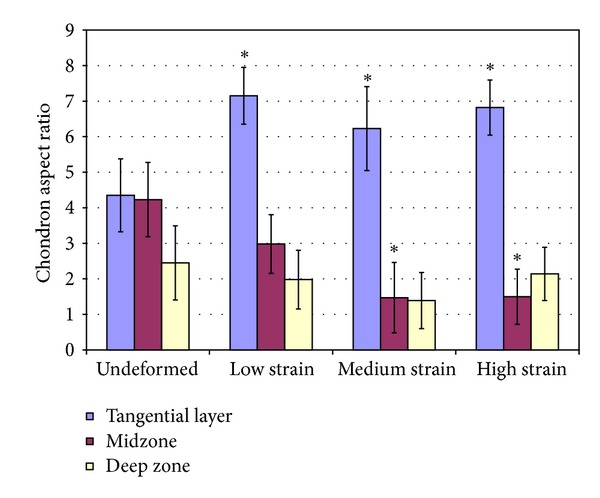
Chondron aspect ratios (see Figure 3) measured for the three different zonal depths and for the three strain levels used. Relative to the undeformed ratios, there were significant differences in the tangential zone for all three strain levels and in the midzone for only the medium and high strain levels (*P* value < 0.05, noted with an asterisk). Compared with the undeformed, there were no significant changes in the aspect ratio of chondrons in the deep zone for all three strain levels.

**Figure 6:**
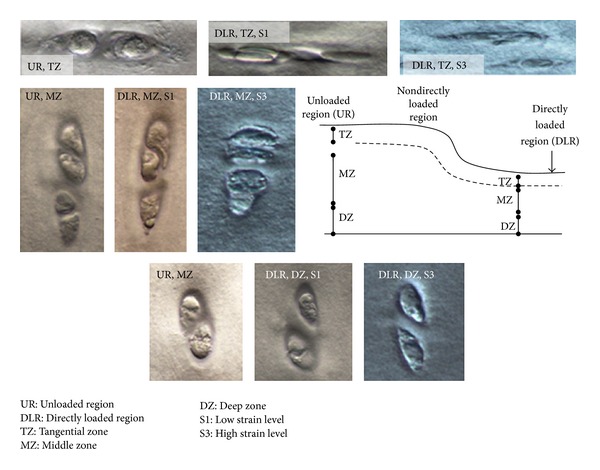
Images of chondrons obtained at different zonal depths in the cartilage matrix, comparing the directly loaded region (DLR) with the nonloaded region (UR). Only in the tangential zone (TZ) are the chondrons deformed at all the levels of applied strain. In the midzone (MZ), there is compression of the chondrons at the applied medium and high strain only. The deep zone (DZ) chondron shape appears unchanged for all the levels of strain.

The chondron aspect ratio in the undeformed cartilage was highest in the tangential and midzones and lowest in the deep zone (Figure 5). The chondron aspect ratio in the tangential layer increased significantly at all strain levels by about 60%. From the DIC images, the increase in aspect ratio was more a result of a large decrease in the short axis of the chondron (see Figure 6).

In the midzone matrix, only the medium and high levels strain produced significant changes in chondron aspect ratio (Figures 5 and 6). These changes resulted from a reduction in height coupled with lateral expansion which together gave aspect ratios that were about 65% smaller than those in the unloaded region. A comparison with the undeformed matrix indicated that in the directly loaded deep zone matrix there was no significant change in the chondron aspect ratio at all levels of strain (Figures 5 and 6). 

**Figure 7:**
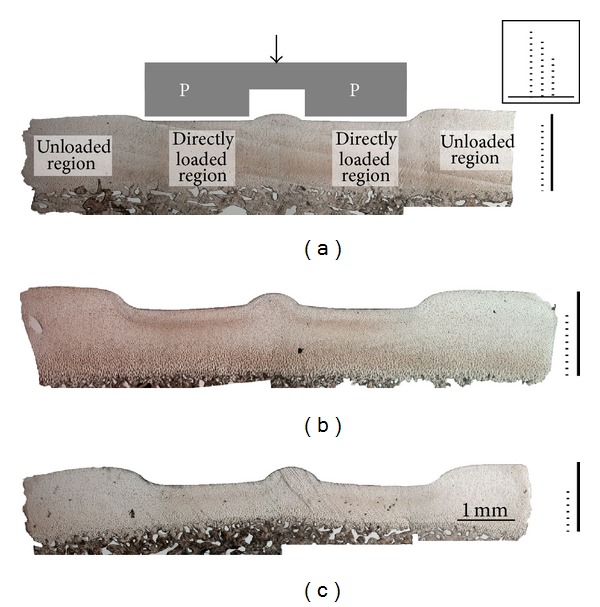
Bulge development resulting from the channel relief zone in the indenter (outlined in grey) between the two directly loaded regions (P). Macroimages (a), (b), and (c) show typical indentation profiles of samples compressed to low, medium, and high levels of strain. The bold and dotted lines on the right gauge the undeformed and deformed thicknesses, respectively, of each of the sections shown. In the boxed inset the dotted lines are shown side-by-side to illustrate the relative differences in the low, medium, and high strain levels in the directly loaded regions.

**Figure 8:**
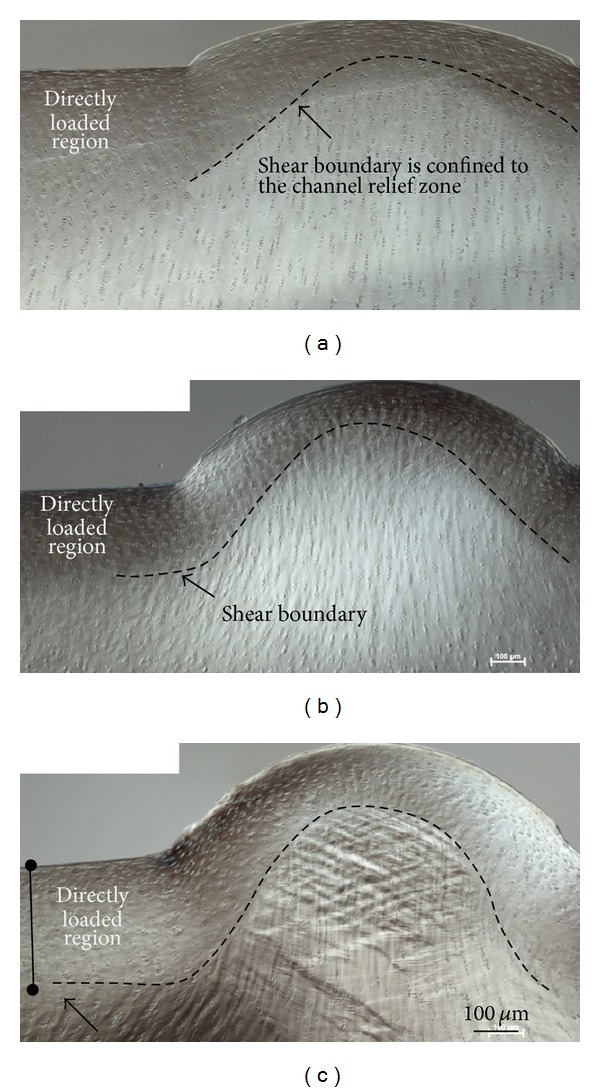
Bulge formation is shown in (a), (b), and (c) for samples tested at the low, medium, and high strain levels respectively. Arrows point to shear boundaries that demarcate differences in microstructural orientation.

The channel relief zone (Figure 7) showed a distinct morphology for each strain level. Specifically the level of matrix extrusion (i.e., the extent of bulge formation) increased progressively with increasing strain. The transition from the directly loaded region to the channel relief zone revealed the extent of shear for each strain level (Figure 8). At the low strain level the shear boundary (see dotted lines in Figures 8(a)–8(c)) was confined to the channel relief zone, whereas at the medium and high strain levels the boundary extended into the directly loaded regions (see dotted lines Figure 8). At the medium strain level in the directly loaded region the shear boundary was located nearer the articular surface, whereas at the high strain level it formed at a greater depth (compare Figure 8(b) with 8(c)). 

**Figure 9:**
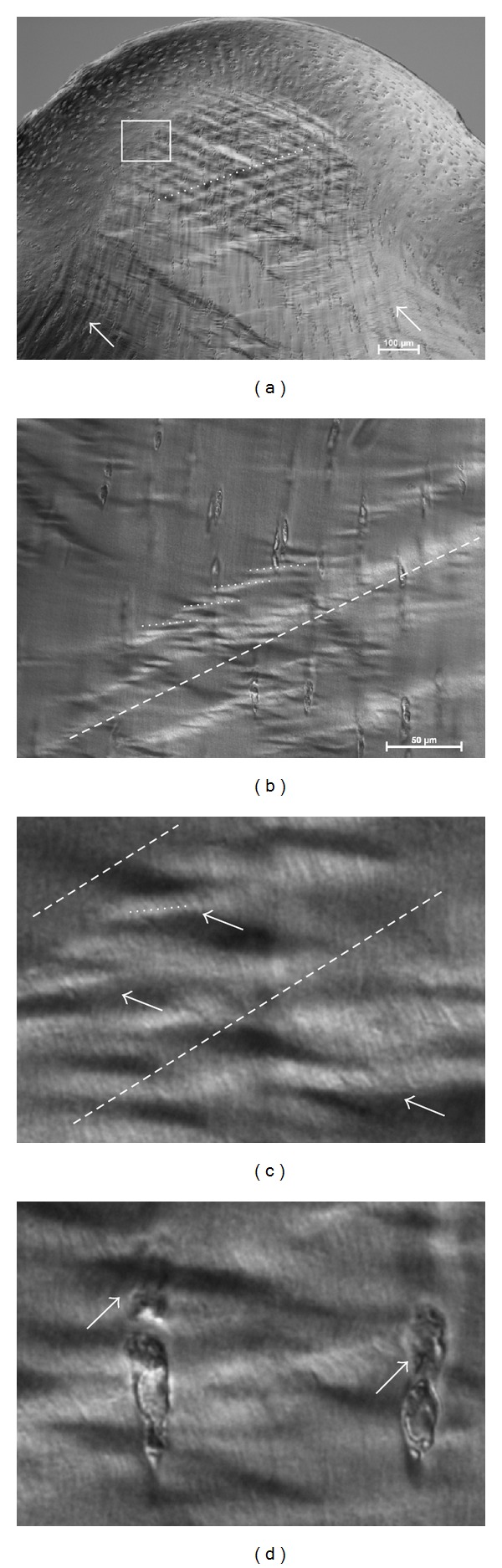
Images of the channel relief zone from a sample tested at the high strain level. (a) Obliquely directed shear bands (dotted line). White arrows in (a) indicate matrix “flow lines” originating from the adjacent directly loaded regions. (b) Smaller dotted lines highlight a transverse directionality to minor compression bands formed within the oblique shear bands (dashed line). (c) shows in greater detail the features of the minor bands within the larger shear bands. Arrows indicate an in-phase fibrillar crimping from which the transversely directed minor bands (highlighted with the small dotted line) are derived. (d) Arrows point to chondrocyte distortion within the transverse bands.

**Figure 10:**
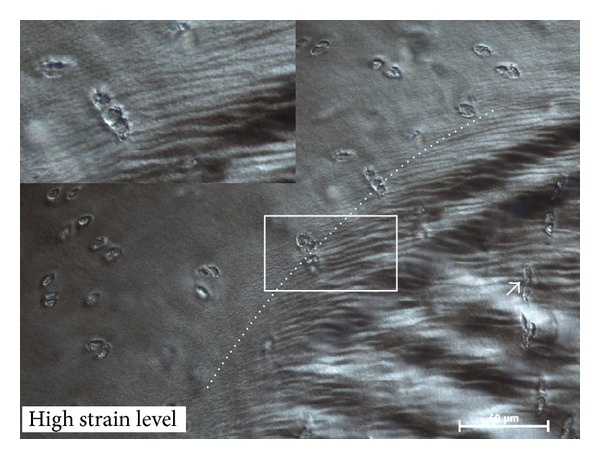
High magnification of boxed region shown in Figure 9(a). The shear bands terminate abruptly at a distinct curvilinear boundary (dotted line). Beyond this boundary the chondrocytes are relatively undistorted by shear (see inset) compared to those within the sheared region (white arrow).

The high strain samples also displayed two oblique sets of intersecting shear bands (at ~45°) in the bulge region (Figure 9(a)). Importantly, these oblique bands were superimposed on strong radial “flow lines” (see white arrows pointing to curvilinear lines in Figure 9(a)) suggesting matrix movement both radially from the deep zone and laterally from both sides of the directly loaded regions. Between the obliquely-directed lines there were minor horizontal bands with a periodicity of about 10 *μ*m. At higher magnification these minor bands were shown to be the result of repeating crests of radial fibres exhibiting an in-phase crimp (Figures 9(b) and 9(c)). Chondrocytes within these minor bands were severely distorted (Figure 9(d)), this distortion disappearing at the distinct boundary defined by the shear band termination (see insert in Figure 10). 

**Figure 11:**
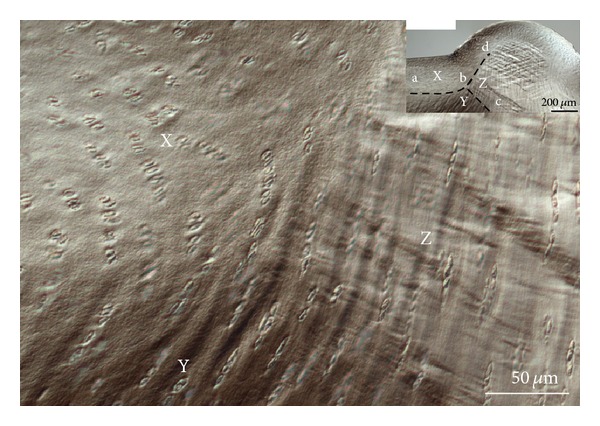
A structural “triple point” is developed between the directly loaded region and the relief zone, and this feature arises from the confluence of three complex deformation fields. One boundary is formed in the directly loaded region between the upper more strain-limiting layer and its underlying matrix (see inset a-b) and defines the chevron shear boundary. The other boundary (c-b-d) separates the relief zone with its oblique shear band development from the upper and lower zones devoid of such oblique shear bands. This triple point highlights the discontinuities in the deformation field associated with the transition between directly and nondirectly loaded regions and across zonal depths. X, Y, and Z show in high magnification the three regions of interest.

In between the directly loaded region and the relief zone there was an interesting point of confluence of three contrasting deformation fields (Figure 11). Previously described as a “triple point” [10] this unique pattern of deformation also showed how abrupt the shear was within the directly loaded region as well as between directly loaded and nondirectly loaded regions (refer to regions X, Y, and Z in Figure 11).

## 4. Discussion

This study seeks to integrate two important characteristics of articular cartilage, namely, its zonally related microstructural response to loading and its nonlinear stress relaxation behaviour. While we have used a simple Maxwell model to describe the stress relaxation behaviour of cartilage-on-bone it should be noted that much more sophisticated theoretical models, such as those developed by Nguyen and Oloyede [17], Julkunen et al. [18], Li et al., [19], and Brown et al. [20], have sought to provide a more comprehensive account of cartilage mechanics. By integrating mechanical and structural responses our aim was to explore how the complex microarchitecture of articular cartilage influences its load-bearing behaviour. 

Our data show that there is a depth-related mechanical response, such that the stress relaxation following different levels of rapid compression can be correlated with the microlevel pattern of deformation. The microstructural images indicate that at low strains only the tangential layer is involved in resisting deformation while increasing the strain from low to medium results in an increased stiffness that is accompanied, structurally, by a visible engagement of the mid-zone matrix. With the additional stress required to increase the strain level from medium to high, the next region of matrix affected is that situated mostly laterally and in the nondirectly loaded region, as evident from the intense shear band formation in the channel relief zone (Figure 8(c)).

By contrast, the deep matrix deformation, as indicated by the chondron aspect ratios, remains relatively uninvolved at all levels of strain. Such an interpretation, however, is limited by the findings in a previous study that reported a nonlinear relationship between cellular-level strains and local extracellular matrix (ECM) strains [4]. Specifically, it was found that in conditions of high local ECM strain the apparent cellular strains appeared to be lower than that in the ECM [4]. Thus, since ECM strains were not measured in the present study, the apparent lack of chondron deformation in the deep zone may not truly represent matrix deformation. However, based on the observed characteristics of the deformation field under the indenter (Figure 8), there is a gradual redistribution laterally of the applied compressive load with increasing radial depth, and this progressive attenuation of stress with increasing radial depth may be an important factor contributing to the lack of measured deformation in the deep zone at the high level of strain. 

As would be expected, the tissue bulge in the relief zone is more pronounced at the medium and high strain levels compared to that in the lowest strain. However, the bulge curvature did not change significantly between the medium and high strain levels—rather there was an increased intensity of oblique and counter-oblique shear band formation in the latter. This suggests that while the maximum effect of the strain limiting surface in resisting the deformation may have already been reached at the medium level of strain, there is increasing matrix consolidation above this medium strain level involving more complex modes of deformation (see Figure 8) as well as relatively abrupt shear discontinuities leading to the formation of a “triple point” in the matrix deformation pattern (see Figure 11). 

It is worth noting that this “triple point,” situated in the directly loaded region near the edge of the channel space, contains a distinct shear discontinuity between the upper and deeper zones that has previously been shown to give rise to the “chevron” pattern of deformation induced during matrix compression [8, 16, 21]. At this same triple point there is a confluence of three different chondrocyte alignments (see regions X, Y, and Z in Figure 11) resulting from the chevron deformation field meeting the radially aligned matrix in the relief zone. The shear bands produced by this confluence are most intense where the shear discontinuity is present in the upper section of the matrix bulge. Further, shear band formation, occurring mostly in the mid to deeper zone matrix (Figure 9), is a clear indication of a significant transverse interconnectivity in the fibrillar structure which helps limit the extent of upwelling of the tissue in the relief zone while also facilitating lateral redistribution of load within the cartilage matrix. 

**Figure 12:**
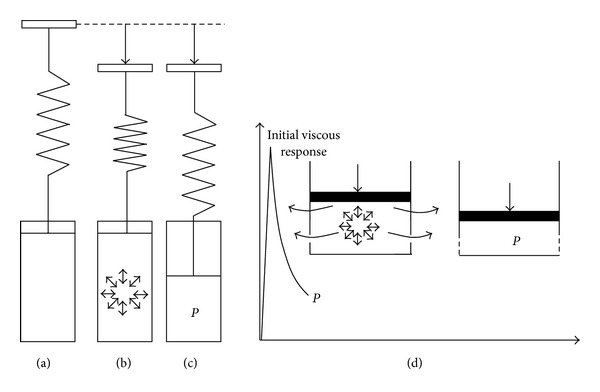
The initial stress relaxation response, or viscous response, is illustrated here. Going from (a) to (b), the initial linear elastic resistance to compression is a combination of the deformation in the spring component and an instantaneous hydrostatic pressure in the dashpot. Going from (b) to (c) the stress then decays, with fluid flow through permeable walls (d), until the quasi-equilibrium state “*P*” is attained following reduction in matrix permeability. This equilibrium stress state *P* is a transition before the next stress relaxation phase takes place (see Figure 13).

Importantly too is the apparent relationship between this lateral redistribution of the load and the stress relaxation response reported for the different strain levels. At all three predetermined strain levels used, because of the relatively rapid rate of loading employed (1 mm/sec), the initial response of the cartilage matrix will approximate near instantaneous, fluid flow-independent elastic behaviour. The initial phase of stress relaxation, that is, the viscous response (see schematics in Figure 12) will be achieved via the outflow of the bound matrix fluid through the initially maximally hydrated collagen/proteoglycan network. Importantly this outflow is via a lateral displacement of fluid given that the indenter is nonporous and the varying levels of lateral deformation of the matrix as shown in the microstructure data. With increasing levels of strain, the matrix becomes more compact the permeability is reduced, and this would then account for the increasing relaxation times (*τ*_1_) recorded for the viscous phase of the stress relaxation response. 

By increasing the level of strain from low to medium to high, the cartilage matrix progressively consolidates, and the viscous response is correspondingly reduced, as direct solid load-bearing increases. This explains why at the lowest strain level there was a larger viscous response without any detectable mid-to-deep zone matrix deformation. Therefore, it is likely that the final near-equilibrium stress at the low strain level reflects *mostly* the matrix swelling pressure generated by the increased concentration of proteoglycans associated with this lowest state of strain. By contrast, at the highest strain levels the equilibrium stress is a measure mostly of the elastic resistance of the near-consolidated solid matrix, that is, one in which the bulk of the moveable fluid has been removed [22]. The equilibrium stress at low strains might therefore be utilised as an indicator of the internal swelling potential and thus potentially useful in assessing matrix changes associated with the early stages of degeneration. These early changes would include both a loss of integrity of the water-binding proteoglycan component and the reduction in their efficiency of entrapment associated with fibril destructuring as described earlier by Broom et al. [23]. 

**Figure 13:**
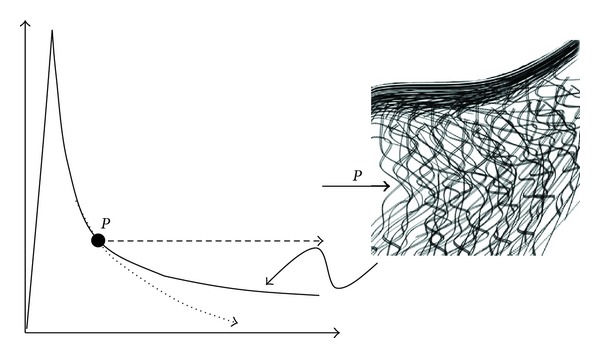
Following the initial stress relaxation response (or viscous response described in Figure 12), the second phase elastic response is illustrated here. The dissipation of stress *P* to a lower stress level and final equilibrium is governed by the solid matrix deformation, laterally. The extent of lateral matrix deformation, as shown in the earlier microimages of increasing shear (Figure 8), is correlated with larger compressive strain levels. Thus the lateral matrix deformation is a function of “*P*” or the amount pressure remaining following the completion of the initial viscous response.

What about the second phase of stress relaxation, that is, the relatively elastic response? It is proposed that this second relaxation phase represents the viscoelastic response of the increasingly consolidated “solid matrix” undergoing deformation as a result of fluid displacement into the nondirectly loaded region (Figure 13) (the term “solid matrix” used in this context refers to the solid components (primarily collagen and PGs) and any still-bound fluid remaining under the prevailing near-equilibrium stress). Such an interpretation is supported by comparing stress relaxation responses from confined compression using a porous indenter (such as that used by Soltz and Ateshian, 1998) with unconfined compression using a nonporous indenter (such as that used both in the present study and by [24]). The stress relaxation curves reported by Soltz and Ateshian [25] do not show the second phase elastic response, while the responses of Mäkelä et al. [24] are very similar to those in the present study. This difference in the stress relaxation curve response could therefore be attributed to a confined versus nonconfined test protocol where, in the latter, lateral flow of fluid can take place into the surrounding matrix. Mäkelä et al. [24] also compared the stress relaxation responses of healthy versus degenerate cartilage and showed that with matrix degeneration the first viscous phase of relaxation occurs very rapidly, followed by a second elastic phase exhibiting minimal further decay in stress over time. 

Active fluid flow processes cannot of course be visualised, only their consequences leading to the near-equilibrium states captured by chemical fixation of the near-equilibrium loaded state. However, our “channel indenter” experiment provides a means of capturing morphologically this complex interplay between structure, mechanical response, and fluid flow in the cartilage-on-bone system. In effect the channel space permits the cartilage throughout its various zonal regions to deform in a manner governed entirely by the restrictions imposed (i) by the strain-limiting tangential zone, (ii) the anchorage into the subchondral plate, (iii) the interconnectivity of the fibrillar network, and (iv) the intrinsic swelling potential constrained within a deformable fibrillar network. 

To conclude, many of the current computationally based models of cartilage are limited by the degree of structural simplification required. It is hoped that our microstructural findings, linked directly to measurable mechanical responses, will contribute to the ongoing development of cartilage models that incorporate higher degrees of structural realism.
